# Severe COVID-19, Another Piece in the Puzzle of the Hyperferritinemic Syndrome. An Immunomodulatory Perspective to Alleviate the Storm

**DOI:** 10.3389/fimmu.2020.01130

**Published:** 2020-05-28

**Authors:** Piero Ruscitti, Onorina Berardicurti, Paola Di Benedetto, Paola Cipriani, Annamaria Iagnocco, Yehuda Shoenfeld, Roberto Giacomelli

**Affiliations:** ^1^Division of Rheumatology, Department of Biotechnological and Applied Clinical Sciences, University of L'Aquila, L'Aquila, Italy; ^2^Academic Rheumatology Centre, Università degli Studi di Torino, Turin, Italy; ^3^Zabludowicz Center for Autoimmune Diseases, Sheba Medical Center, Tel HaShomer, Ramat Gan, Israel; ^4^Sackler Faculty of Medicine, Tel-Aviv University, Tel Aviv-Yafo, Israel; ^5^Laboratory of the Mosaics of Autoimmunity, Saint Petersburg State University, Saint Petersburg, Russia

**Keywords:** COVID-19, hyperferritinemia, hyper-inflammation, hyperferritinaemic syndrome, adult onset Still's disease, haemophagocytic lymphohistiocytosis, catastrophic anti-phospholipid syndrome

## Abstract

The coronavirus disease 2019 (COVID-19), an acute respiratory disease caused by severe acute respiratory syndrome-coronavirus-2 (SARS-CoV-2), has been declared as a worldwide public health emergency. Interestingly, severe COVID-19 is characterized by fever, hyperferritinemia, and a hyper-inflammatory process with a massive release of pro-inflammatory cytokines, which may be responsible for the high rate of mortality. These findings may advocate for a similarity between severe COVID-19 and some challenging rheumatic diseases, such as adult onset Still's disease, secondary hemophagocytic lymphohistiocytosis, and catastrophic anti-phospholipid syndrome, which have been included in the “hyperferritinemic syndrome” category. Furthermore, as performed in these hyper-inflammatory states, severe COVID-19 may benefit from immunomodulatory therapies.

## Introduction

The coronavirus disease 2019 (COVID-19) is an acute respiratory disease caused by a novel coronavirus (severe acute respiratory syndrome-coronavirus-2, SARS-CoV-2), identified in Wuhan, China in December 2019 (1). Since then, the COVID-19 outbreak has spread worldwide, becoming a pandemic, causing a public health emergency, according to the World Health Organization (WHO), and resulting in thousands of deaths (1). SARS-CoV-2 is a β-coronavirus, an enveloped non-segmented positive-sense RNA virus, which could be transmitted from bats via unknown intermediate hosts to infect humans, using the angiotensin-converting enzyme 2 (ACE2) receptor (2). The latter, is more expressed in adults than children, and thus possibly explains why the disease is more aggressive in older patients (3). COVID-19 shows a heterogeneous course, from patients affected by mild flu-like symptoms to patients with unremitting fever and severe respiratory involvement. On this basis, markers of poor prognosis have recently been investigated to effectively prioritize resources to patients with more severe symptoms (4). Interestingly, this study identified hyperferritinemia and interleukin (IL)-6, as predictors of poor outcome, thus suggesting a hyper-inflammatory process as the major cause of death (4, 5). In severe COVID-19, a specific cytokine profile resembling the pattern of a secondary hemophagocytic lymphohistiocytosis (HLH) has been shown, due to significant increases of IL-2, IL-7, granulocyte colony stimulating factor (GCSF), interferon-γ inducible protein 10 (IP-10), monocyte chemoattractant protein 1 (MCP-1), macrophage inflammatory protein 1-α, and tumor necrosis factor (TNF) (6). Contextualizing unremitting fever, hyperferritinemia, and the hyper-inflammatory process, severe COVID-19 shows similarity to disorders comprised in the so-called hyperferritinemic syndrome (7). This syndrome includes adult onset Still's disease (AOSD), systemic juvenile idiopathic arthritis (SJIA), secondary HLH, catastrophic anti-phospholipid syndrome (cAPS), and septic shock (7). Hyperferritinemia is a common trait of all these forms, which could be an active pathogenic mediator and not only a consequence of the inflammation (7). On these bases, we aimed to review the similarities between severe COVID-19 and diseases included in hyperferritinemic syndrome, from a pathogenic, clinical, and therapeutic point of view, thus proposing new insights to improve the management of those patients.

## Ferritin as a Pro-Inflammatory Enhancer in Severe COVID-19

Coronavirus RNAs may act as pathogen-associated molecular patterns, which are detected by the pattern recognition receptors and activate downstream cascades pro-inflammatory pathways (1, 6). In the endosome, toll-like receptor (TLR) 3, TLR7, TLR8, and TLR9 may sense viral RNA and DNA (8), whereas, in the cytoplasm, the viral RNA receptor retinoic-acid inducible gene I (9), cytosolic receptor melanoma differentiation-associated gene 5, and nucleotidyltransferase cyclic guanosine monophosphate–adenosine monophosphate (GMP-AMP) synthase may recognize viral RNA and DNA (10). Consequently, downstream cascades molecules are triggered, involving adaptor molecule myeloid differentiation primary response 88 (MyD88), transcription factor nuclear factor-κB (NF-κB), and interferon regulatory factor 3, leading to the production of pro-inflammatory molecules (11, 12). In fact, plasma cytokines and chemokines were increased in COVID-19 patients, including IL-1β, IL-2, IL-4, IL-7, IL-10, IL-12, IL-13, IL-17, GCSF, IP-10, interferon-γ (IFN-γ), and TNF (6). During severe COVID-19, these mechanisms could be exaggerated, probably because of a specific genetic susceptibility (13), and these patients are characterized by very high blood levels of pro-inflammatory mediators and ferritin (4, 5). The latter is an iron-binding molecule, which is produced after pro-inflammatory stimuli, in addition to iron availability (7). Furthermore, ferritin comprises 24 subunits, codified according to their molecular weight into heavy (FeH) and light (FeL) subunits. Remarkably, increased expression of FeH and of CD68+/FeH+ macrophages may be observed in inflammatory infiltrate of AOSD and secondary HLH (14, 15). Additionally, a stimulatory effect of FeH on NF-kB has been described, acting as a pro-inflammatory cytokine on hepatic stellate cells (16). In this work, ferritin was shown to regulate an iron-independent signaling pathway that resulted ultimately in NF-kB activation (16), thus converging on the same pathway elicited by SARS-CoV-2 RNAs (11, 12). On the contrary, the deletion of FeH reduced the inflammatory burden in the model of sepsis by lipopolysaccharide-induced endotoxemia, cecal ligation, and puncture (17). Such protection was predominantly mediated by the compensatory increase in FeL, associated with an inhibitory action on NF-kB (17). The pro-inflammatory cytokines, which are elevated in hyperferritinemic syndrome, have also been described in severe COVID-19 (6), and may preferentially induce the expression of FeH, via FER2, a regulatory element acting as a binding site to NF-kB. The latter, in turn, stimulates the synthesis of further FeH and pro-inflammatory cytokines, thus perpetuating a vicious inflammatory loop (7). In addition, FeH+/IL-12+ macrophages have been shown in the infiltrate of AOSD and secondary HLH (15, 18), which may further release FeH, following inflammatory stimuli (19), and thus contributing to the inflammatory loop (20). On these bases, we hypothesize that severe COVID-19 shares common pathogenic mechanisms with other diseases of hyperferritinemic syndrome (7), with ferritin enhancing the inflammatory burden and triggering a vicious pathogenic loop.

## The Lung, at the Crossroad Between Severe COVID-19 and Hyperferritinemic Syndrome

Lung involvement and hyper-inflammation are at the crossroad between severe COVID-19 and the hyperferritinemic syndrome. As observed in other β-coronaviruses diseases, COVID-19 is characterized by fever, dry cough, increasing dyspnoea with hypoxemia, and bilateral ground-glass opacities and patchy shadowing with a peripheral or posterior distribution, mainly in the lower lobes, on chest CT scans (6). In fact, an anatomy report of a COVID-19 pneumonia cadaver showed that SARS-CoV-2 invades the respiratory mucosa and infects other cells, thus provoking an inflammatory response in the lower airway and causes lung injury (21). Considering that coronavirus binds to the host cells using the ACE2 receptor, which is highly represented in the lower respiratory tract, a persistent and repeated stimulation of TLRs in the lung may occur, hence triggering an aberrant immune response and the production of a cytokine storm (22). The latter is the result of overwhelming systemic inflammation with a massive release of pro-inflammatory cytokines, quickly progressing to multiple organ dysfunction syndrome and eventually to death (23). In spite of various inflammatory etiologies, cytokine release syndrome is supported by an essential underlying hypothesis: the massive release of cytokines as a consequence of: (i) excessive and repeated inflammatory stimuli, and (ii) an inadequate regulation of inflammation, (iii) an uncontrolled release of cytoplasmic cytokines from destroyed lymphocytes after anti-cancer therapies (23, 24). In addition, it has been shown that increased amounts of pro-inflammatory cytokines, including IL-1β, IL-6, IL-12, IFN-γ, IP-10, and MCP1, were associated with pulmonary inflammation and extensive lung damage in SARS patients (25), thus suggesting a further pathogenic loop in inducing the cytokine storm. Although the mechanisms of how COVID-19 and, in other more general viral infections, would prompt the cytokine storm syndrome are not fully elucidated, it has been suggested that the IFN-γ, which is largely released by a variety of hematopoietic cells in response to viral infection, may facilitate the occurrence of hyper-inflammation (23). In patients with SJIA, lung involvement may trigger systemic inflammation and the development of secondary HLH and IFN-γ plays a central pathogenic role (26, 27). In fact, in lung biopsies in patients with SIJA, the analysis of expressed genes revealed that many of the up-regulated targets were in gene pathways related to an IFN-γ signature, including human leukocyte antigen (HLA)–D family members and other IFN-related genes (26, 27). Two of the most highly up-regulated non-HLA genes were chemokine (C-X-C motif) CXCL9, and CXCL10 (26), which are IFN-induced chemokines strongly correlated with the occurrence of secondary HLH (28). In addition, the lung is one of the major physiological producers of IL-1β and IL-6 (29), which are also involved in pathogenic steps, leading to the occurrence of secondary HLH (30, 31). Considering all of these findings, it is possible to postulate that during the acute respiratory distress syndrome of COVID-19, the SARS-CoV-2 may trigger a hyper-inflammatory reaction strongly resembling that observed in the lung involvement of SJIA, in which the lung acts as a trigger to amplify the immune response. The final result is the uncontrolled proliferation of activated immune cells, the massive production of pro-inflammatory mediators, and the development of cytokine storm syndrome, either in severe COVID-19 or SJIA.

## Unremitting Fever and Hyperferritinemia as Common Clinical Traits Between Severe COVID-19 and Hyperferritinemic Syndrome

From a clinical point of view, severe COVID-19 and the diseases included in hyperferritinemic syndrome share a fever as the main clinical symptom. In these conditions, the analysis of fever pattern would also suggest a useful clue to assess the severity of the disease and the occurrence of complications. In SJIA and AOSD, a typical change from the high-spiking intermittent typical quotidian pattern, to a continuous unremitting pattern suggests the occurrence of secondary HLH, and the worsening of the clinical situation toward a life-threatening hyper-inflammatory complication (32). During COVID-19, on the basis of observations from clinicians on the frontlines, the occurrence of unremitting fever would similarly identify a more aggressive subset of patients, at higher risk of a poor prognosis. In addition, in severe COVID-19, hyperferritinemia is observed, suggesting a marker of severity (4, 5). Although it has poor specificity, a 5-fould increase of ferritin is strongly suggestive of the diseases included in hyperferritinemic syndrome, and is a useful marker to assess disease activity and to predict a poor prognosis (20). In fact, hyperferritinemia is associated with increased mortality in sepsis, multiple organ dysfunction syndrome, and critical illness (33–35). Thus, the clinical phenotype, characterized by unremitting fever and hyperferritinemia, identifies the most severe subset of COVID-19 as observed in the diseases included in hyperferritinemic syndrome.

## An Immunomodulatory Therapeutic Perspective in COVID-19

**Figure 1:**
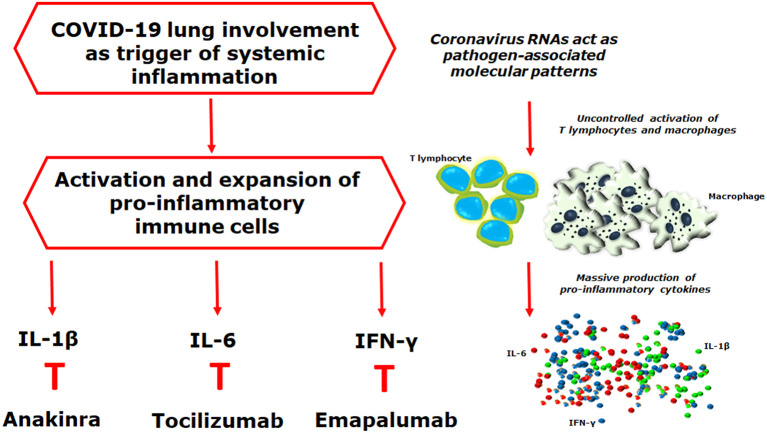
Coronavirus RNAs may act as pathogen-associated molecular patterns, detected by the pattern recognition receptors, triggering downstream cascades molecules, and leading to the production of pro-inflammatory mediators. Interestingly, during the acute respiratory distress syndrome of coronavirus disease 2019 (COVID-19), the severe acute respiratory syndrome-coronavirus-2 (SARS-CoV-2) may trigger a hyper-inflammatory reaction strongly resembling what observed in the lung involvement of systemic juvenile idiopathic arthritis, in which the development of a pulmonary hyper-inflammatory process, has been reported, mediated by an increased release of interleukin (IL)-1β and IL-6, associated with a tissue hyper-expression of interferon (IFN)-related genes. The final result of all these mechanisms is the uncontrolled proliferation of activated immune cells, the massive production of pro-inflammatory mediators, and the development of cytokine storm syndrome. On these bases, it is reasonable to postulate the clinical usefulness of IL-1 β, IL-6, and IFN-y inhibition on targeting severe COVID-19, as reported in other hyper-inflammatory diseases.

Considering the lack of efficacy of antiviral therapy for severe coronavirus infection, it is reasonable to postulate the clinical usefulness of specific immunomodulatory therapies (Figure 1), as observed for other diseases included in hyperferritinemic syndrome such as intravenous immunoglobulins (IVIGs) and tocilizumab, the humanized monoclonal antibody against IL-6 receptor (7). *Ex juvantibus*, one of the best criteria for identifying a common pathogenic mechanism, among different diseases, is that the clinical manifestations were reversed upon initiation of the same therapy. It has been shown that, after IVIGs therapy, a significant reduction of hyperferritinemia, both in sepsis and secondary HLH was observed, correlating with an improvement in patients (7). Considering their proposed anti-viral activity, possibly comprising many cross-reacting anti-viral antibodies and *per se* immunomodulatory activities (36), IVIGs has also been proposed to treat severe COVID-19 (37). Another therapeutic immunomodulatory possibility in severe COVID-19 is the administration of hydroxychloroquine (HCQ). This drug, has been a licensed treatment for rheumatoid arthritis for many years, and was shown to reduce the viral load, favoring the disappearance of SARS-CoV-2 (38). However, although it seems promising, a recent meta-analysis, including 1,358 patients, suggested that more data are required for a definitive conclusion on the use of HCQ in this setting, since no difference was observed in virologic cure, death, or clinical worsening of disease between HCQ-treated patients and control groups (39). As far as tocilizumab is concerned, the rationale for its use in severe COVID-19 derived from evidence of its beneficial effect on cytokine release syndrome. This is a clinically significant, on-target, off-tumor side effect of the chimeric antigen receptor T-cell therapies administered for treatment of malignancies (24). Characteristics of cytokine-release syndrome include fever, encephalopathy, hypotension, and coagulopathy, leading to multiorgan failure, associated with very pronounced levels of hyperferritinemia and IL-6 (24). The latter provided an effective therapeutic target in cytokine release syndrome (40). Mirroring this finding, tocilizumab has been used to treat severe COVID-19 with promising results, as observed in other diseases of hyperferritinemic syndrome (7). Furthermore, a reduction of ferritin, obtained by combing immunomodulatory drugs, was associated with a lower mortality rate in cAPS and HLH (7), thus possibly suggesting the use of, in a more aggressive subset of COVID-19, a combination therapy with both antiviral and anti-inflammatory drugs, at the same time (41). In addition, the repurposing of these drugs in severe COVID-19 could benefit from the findings of previous reports, and thus, on this basis, many clinical trials are ongoing in different countries (ChiCTR2000029765, NCT04317092, NCT04310228, and NCT04332913).

As far as other immunomodulatory strategies in COVID-19 are concerned, IL-1 inhibition showed benefits in sepsis, in which both hyperferritinemia and hyper-inflammation, may be observed, contributing to the dysregulation of the host immune system (42). A *post-hoc* analysis of data from a phase 3 randomized controlled trial showed some improvement of patients with sepsis, following anakinra, a recombinant non-glycosylated form of human IL-1 receptor antagonist, thus suggesting its possible use in those patients (42). As a consequence, it is possible to hypothesize that anakinra may also relieve severe COVID-19. Reported data suggest the possible efficacy of emapalumab, a monoclonal antibody neutralizing IFN-y, approved in the treatment of HLH and its massive production of pro-inflammatory cytokines (43). Due to the important role of IFN-y in driving hyper-inflammation during viral infections, emapalumab may be an additional immunomodulatory therapy that could be employed in the treatment of severe COVID-19. In addition, available literature suggests that janus kinase (JAK) inhibition might affect COVID-19 twice as much, by targeting both inflammation and cellular viral entry (44). It has been proposed that baricitinib, a JAK1/JAK2 inhibitor, may control the hyper-inflammatory steps in those diseases, characterized by a cytokine storm, since a plethora of cytokine receptors indiscriminately use these JAKs as mediators of ligands binding and consequent activation of the inflammatory cascade (45). Furthermore, the disruption of P2-associated protein kinase 1, a known regulator of viral endocytosis into the cell, by baricitinib, could possibly be an additional positive effect in COVID-19, decreasing the viral entry (44). Finally, considering ferritin as a pathogenic mediator, this could also be proposed as a therapeutic target in these conditions. High-volume hemofiltration and plasma exchange, extracorporeal blood purification techniques, have been employed to treat secondary HLH to sepsis (46–48). Interestingly, in parallel with the clinical efficacy, these procedures induce a ferritin reduction (46–48), suggesting that the mechanical removal of ferritin could have a possible therapeutic role.

## Discussion

In this work, we discuss the similarities, from a pathogenic, clinical, and therapeutic point of view, between severe COVID-19 and four conditions; secondary HLH, AOSD, cAPS and septic shock, which are included in hyperferritinemic syndrome. All these diseases are characterized by very high levels of ferritin, which could not only be the product of the inflammation but rather may play a pathogenic role. Possibly, in an inflammatory environment, as observed in these diseases, hyperferritinemia may be involved in a vicious pathogenic loop prompting its pro-inflammatory properties. In severe COVID-19, ferritin could be a further possible enhancer of the cytokine storm. Clinically, unremitting fever is a common feature of severe COVID-19, suggesting that a change from the intermittent quotidian pattern to a continuous unremitting form would indicate a worsening toward the cytokine storm, as in AOSD and SJIA. The hyperferritinemia seems to be a marker of poor prognosis and response to treatment, in both severe COVID-19 and hyperferritinemic syndrome. Finally, the good response to immunomodulatory therapies, observed during severe COVID-19, strongly supports the link between this form and other diseases included in hyperferritinemic syndrome. In addition, targeting the hyper-inflammatory process, through immunomodulatory therapies, decreases the high mortality rate of all these diseases (7, 49–51), thus proposing additional therapeutic options to improve the survival of severe COVID-19 patients, the latter characterized by an over-exuberant pro-inflammatory response, in which the viral load is not correlated with the worsening of symptoms (6).

In conclusion, we hypothesize that severe COVID-19 shares pathogenic mechanisms, a clinical picture, outcomes, and therapeutic strategies with disorders included in hyperferritinemic syndrome. The hyperferritinemia, characterizing all these diseases may be a pathogenic mediator, enhancing the inflammatory burden, and, as observed in AOSD, cAPS, and secondary HLH, its reduction is associated with a lower mortality. Thus, at present, severe COVID-19, seems to be a new entity in hyperferritinemic syndrome. In addition, since accumulating evidence suggests that severe COVID-19 is associated with a cytokine storm syndrome, therapeutic strategies combining immunomodulatory therapies, may improve the management of those patients. Furthermore, in this setting, high levels of ferritin, identifying a more aggressive subset of COVID-19, may drive clinicians to apply more aggressive therapies and resources in those patients, thus balancing appropriate escalation of therapy and minimizing the exposure to iatrogenic harm. SARS-CoV-2 and consequent COVID-19 are a new and great challenge for health systems worldwide, requiring a multidisciplinary approach and a large body of knowledge.

## Author Contributions

All the authors meet all criteria for authorship in the ICMJE recommendations, since all authors made substantial contributions to the conception or design of the work, the acquisition and interpretation of data. All authors contributed to the critical review and revision of the manuscript and approved the final version. All the authors agreed to be accountable for all aspects of the work.

## Conflict of Interest

The authors declare that the research was conducted in the absence of any commercial or financial relationships that could be construed as a potential conflict of interest.
